# Impact of Kalanchoe *(Kalanchoe daigremontiana*) Supplementation in Goat Maternal Diet on Hepatic and Renal Function and Reproductive Performance †

**DOI:** 10.3390/biology14040376

**Published:** 2025-04-05

**Authors:** Juan M. Vázquez-García, Gilberto Ballesteros-Rodea, Venancio Cuevas-Reyes, Luisa E. S. Hernández-Arteaga, Luz Y. Peña-Avelino, Samuel López-Aguirre, Reagan Sims, Jaime M. Cavazos-Galindo, Cesar A. Rosales-Nieto

**Affiliations:** 1 Facultad de Agronomía y Veterinaria, Universidad Autónoma San Luis Potosí, San Luis Potosí 78321, Mexico; manuel.vazquez@uaslp.mx (J.M.V.-G.); gilberto.ballesteros@uaslp.mx (G.B.-R.); socorro.hernandez@uaslp.mx (L.E.S.H.-A.); 2 Instituto Nacional de Investigaciones Forestales, Agrícolas y Pecuarias, Campo Experimental Valle de México, Texcoco 56250, Mexico; cuevas.venancio@inifap.gob.mx; 3 Facultad de Medicina Veterinaria y Zootecnia, Universidad Autónoma de Tamaulipas, Ciudad Victoria 87000, Mexico; lypena@docentes.uat.edu.mx; 4 Facultad de Medicina Veterinaria y Zootecnia, Universidad Veracruzana, Veracruz 91710, Mexico; samuellopez@uv.mx; 5 Department of Agricultural Sciences, Texas State University, San Marcos, TX 78666, USA; r_sims@txstate.edu; 6 Centro de Fomento Ganadero Vallecillo, Universidad Autónoma de Nuevo León, San Nicolás de los Garza 65415, Mexico; jaime_cavazos76@hotmail.com

**Keywords:** medicinal plant, liver enzymes, kidney function, metabolite markers, Alpine goats

## Abstract

*Kalanchoe daigremontiana*, a medicinal plant rich in bioactive compounds, has the potential to serve as a feed supplement, reducing reliance on conventional livestock medications while potentially enhancing productivity. This study evaluated the effects of *K. daigremontiana* supplementation over 52 days on kidney and liver function, metabolism, weight changes, and reproductive efficiency in multiparous Alpine goats during the breeding season. Fifty-five goats were assigned to either a control (CTL, n = 27; 47.6 ± 1.1 kg) or a *K. daigremontiana*-supplemented (KAL, n = 28; 47.6 ± 1.3 kg) diet. The KAL group received *K. daigremontiana* at a dry matter-based inclusion rate of 2 kg t^−1^ of feed, while the CTL group received no supplementation. Blood samples were collected at four time points to assess the biochemical markers of kidney and liver function. Pregnancy was achieved through natural mating, and reproductive efficiency was evaluated. Overall, liver and kidney function did not differ significantly between treatments (*p* > 0.05). However, KAL supplementation was associated with increased creatinine (*p* < 0.05), ALP (*p* < 0.001), and bilirubin (*p* < 0.05) at specific time points, whereas CTL goats exhibited higher BUN (*p* < 0.001), AST (*p* < 0.05), albumin (*p* < 0.001), total protein (*p* ≤ 0.05), and phosphorus (*p* < 0.01) on specific dates. Other metabolic markers, weight gain, and reproductive efficiency did not differ between treatments (*p* > 0.05). Overall, *Kalanchoe daigremontiana* supplementation had mild, transient effects on goat health without significantly impacting productivity. Further research is needed to explore its long-term effects and optimal dosage for livestock nutrition.

## Simple Summary

*Kalanchoe daigremontiana*, a medicinal plant rich in bioactive compounds, was evaluated as a potential livestock feed supplement to enhance production efficiency. This study assessed the effects of *Kalanchoe daigremontiana* supplementation on kidney and liver function, metabolism, weight, and reproductive performance in 55 multiparous Alpine goats over a 52-day period. Although some metabolic differences were observed between treatment groups, overall liver and kidney function, weight gain, and reproductive performance remained similar. The findings suggest that *Kalanchoe daigremontiana* supplementation has mild, transient effects on goat health, warranting further research on long-term impacts and optimal dosing.

## 1. Introduction

The growing demand for more productive and sustainable food production systems, combined with the high cost of animal feed—particularly during the off-season—has led to increased interest in alternative feed sources for livestock. Medicinal plants have gained attention in animal nutrition due to their potential health benefits and cost-effectiveness [1,2]. *Kalanchoe daigremontiana* (*K. daigremontiana*) is widely recognized for its medicinal properties [3] and its specialized crassulacean acid metabolism (CAM) photosynthetic system, which enhances biomass production efficiency and optimizes water use [4]. Its ability to retain moisture and nutrients allows it to thrive in nutrient-poor soils and drought conditions, making it well-suited for semi-arid and arid environments [5]. Due to its resilience, *K. daigremontiana* is widely distributed across temperate regions of Latin America, Asia, and Africa [6,7,8].

Our recent findings indicate that *K. daigremontiana* contains 18% dry matter, 18.9% carbohydrates, 6.6% crude protein, 22.9% crude fiber, and 1.97% ether extract [9]. Additionally, comprehensive analyses have identified a variety of bioactive compounds, including flavonoids, tannins, saponins, alkaloids, glycosides, vitamins, minerals, and antioxidants [10,11,12]. *K. daigremontiana* exhibits antimicrobial and anti-inflammatory properties that may reduce gastrointestinal parasite loads, enhance ATPase activity, and mitigate oxidative stress in reproductive cells and tissues [13,14]. These effects could improve immune function, support pregnancy establishment, and reduce fetal loss. ATPase activity is critical in energy metabolism, and increased energy availability during conception may enhance ovulation rates [15]. During lactation, elevated ATPase activity can improve colostrum quality and increase offspring survival during the perinatal period [16]. The antioxidant content of *K. daigremontiana* may further contribute to livestock productivity. Previous studies suggest that antioxidant supplementation during conception, late pregnancy, and early lactation can enhance reproductive performance and overall production efficiency [17,18,19]. Incorporating *K. daigremontiana* into livestock feed may also reduce reliance on conventional medications and supplements, potentially lowering production costs for goat farmers. However, concerns regarding its toxicity have been raised due to the presence of cardiotoxins, which have led to livestock losses in South Africa and Australia when consumed without sufficient forage availability [20,21]. Notably, goats have demonstrated the ability to integrate toxic plants into their diet under forage-limited conditions without exhibiting symptoms of toxicity or experiencing significant production losses [22,23]. Further research is needed to determine the optimal inclusion rate of *K. daigremontiana* in goat diets to maximize its benefits while minimizing potential risks.

Extensive goat production systems play a critical role in global food security, particularly in semi-arid and arid regions. The sustainability of these systems is heavily dependent on favorable climate conditions; however, climate change is altering climatic patterns, which negatively impacts production [24,25]. Reduced quality or availability of animal feed can induce a catabolic state, leading to fat mobilization, impaired liver function, decreased conception rates, increased fetal losses, and lower productivity in female goats [26,27,28,29]. One potential strategy to mitigate these effects is the inclusion of *K. daigremontiana* in goat diets. The bioactive compounds in *K. daigremontiana* may enhance kidney and liver enzyme function, thereby improving nutrient absorption and utilization. This could improve feed efficiency, growth performance, and reproductive success. While no studies have specifically examined the effects of *K. daigremontiana* on kidney and liver function or reproductive physiology in goats, research in other species suggests potential benefits for growth. For instance, Cobb chickens supplemented with *K. daigremontiana* exhibited higher daily weight gain, improved feed conversion efficiency, greater final weight, and reduced liver enzyme activity [30]. Integrating *K. daigremontiana* into goat diets offers a promising opportunity to enhance animal health and performance, particularly in resource-limited areas, while promoting sustainable feeding practices. However, prior to its inclusion in goat diets, it is essential to determine appropriate dosages, evaluate its effects on kidney and liver function, and assess its overall impact on animal health and productivity to ensure safe and effective use. We hypothesize that supplementing goat diets with *K. daigremontiana* enhances kidney and liver function, improving nutrient absorption, feed efficiency, growth performance, and reproductive success.

## 2. Materials and Methods

This study was conducted during the breeding season (September) at the Faculty of Agronomy and Veterinary Medicine, the Autonomous University of San Luis Potosí (22°13′ N, 100°51′ W). All animal procedures were approved by the university’s Institutional Animal Care and Use Committee (C19-FAI-05-86.86, 511-6/2019.-8024; 511-6/2019.-12305) and adhered to the ARRIVE guidelines for reporting animal research [31], as well as relevant international [32] and national regulations [33]. The local climate is classified as cold desert (BsKw, Köppen classification), with an average annual temperature of 18 °C and an annual precipitation of approximately 341 mm. March is the driest month, with an average of 6 mm of rainfall, while June is the wettest, receiving an average of 67 mm. The photoperiod ranges from 10.5 h of light per day in December to 13.5 h in June [34].

### 2.1. Experimental Design

**Figure 1:**
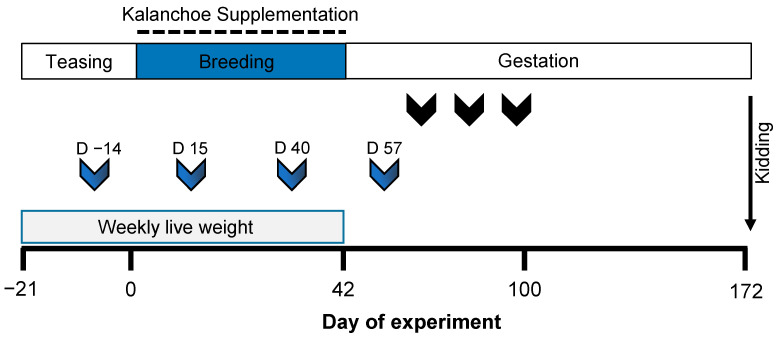
Experimental design of maternal supplementation with *Kalanchoe daigremontiana* during the breeding period in Alpine multiparous goats from a subtropical location (22°13′ N, 100°51′ W). Day 0 represents the start of the *Kalanchoe daigremontiana* supplementation. Prior to breeding, the goats were teased, and pregnancy was subsequently achieved through natural mating with experienced bucks over a 42-day period. Blood samples were collected at four time points: Day 14, Day 15, Day 40, and Day 57 (indicated by blue arrows). Pregnancy status was assessed at three time points, occurring 35–60 days after the removal of the fertile bucks (indicated by black arrows). Weekly live weights were recorded during the teasing and breeding periods.

The experimental protocol is illustrated in Figure 1. In September (the breeding season), 55 multiparous Alpine goats, each with a minimum of three parities and proven fertility, were randomly assigned to one of two dietary treatments while ensuring similar average body weights across groups: Kalanchoe (KAL; n = 28; 47.6 ± 1.3 kg) and control (CTL; n = 27; 47.6 ± 1.1 kg). The goats in the KAL group received a dry matter-based supplement of *K. daigremontiana*, whereas those in the CTL group did not (see details below).

This study aimed to evaluate the effects of dietary *K. daigremontiana* supplementation during the breeding season on kidney function (blood urea nitrogen [BUN] and creatinine), liver function (alanine aminotransferase [ALT], alkaline phosphatase [ALP], aspartate transaminase [AST], albumin, bilirubin, gamma-glutamyl transpeptidase [GGT], and total protein), circulating metabolites (glucose, cholesterol), minerals (phosphorus and calcium levels), body weight changes, and reproductive performance (fertility, prolificacy, and reproductive rate—see details below).

### 2.2. Kalanchoe daigremontiana and Nutritional Diet

Whole *K. daigremontiana* plants were collected from the Desert Zones Research Institute of the Autonomous University of San Luis Potosí, Mexico. A certified professional affiliated with the institute identified a reference specimen. The plants were cleaned and dried in a Felisa 40L FE-291D forced-air oven (Feligneo, S.A. de C.V., Jalisco, Mexico) at 75 °C until a constant weight was achieved. They were then ground to a particle size of 2 mm using a Thomas Wiley Mill (Thomas Scientific LLC, Swedesboro, NJ, USA). The dried and ground material was stored in an amber glass container at room temperature (22 °C) until further use.

A total mixed ration was provided in a feeder with sufficient space (30 cm of linear space per goat) to minimize competition, ensuring free access to feed. Throughout the experiment, diets met the nutritional requirements for mature, non-pregnant animals with limited physical activity [35]. The balanced isoproteic and isoenergetic diet, consisting of maize silage, alfalfa hay, and oat hay, was fed twice daily in equal portions. It contained 8% crude protein (CP) and provided 2.03 Kcal/kg of energy (Table 1). The dried and ground *K. daigremontiana* was added to the diet at 2 kg per ton of feed and administered to the KAL group for 52 days (September–October), including a 10-day adaptation period (late August). The dosage was determined based on a previous study using the same plant in poultry, with adjustments for small ruminants [30]. The CTL group received no additive supplementation. To our knowledge, *K. daigremontiana* has not been previously administered as a dietary supplement in domestic animals. In vitro studies have used a 20 mg/mL dose to evaluate its antioxidant properties and cytotoxicity [36].

The goats were managed according to the goat unit’s methodology, which included deworming (Baymec^®^; Bayer, CDMX, Mexico) and applying multivitamins (Catosal^®^ and Vigantol^®^; Bayer, CDMX, Mexico) before breeding. Throughout this study, the goats had continuous access to clean water and mineral salt blocks.

### 2.3. Reproductive Management and Variables

As part of the goat unit management, the goats were teased before the start of the breeding period (Day -21; [37]) to induce ovulation [38]. Afterwards, during September, pregnancy was achieved through natural mating with experienced bucks over 42 days (two complete reproductive cycles). At the onset of mating (Day 0), the goats were assigned to their respective treatment groups and housed in two breeding pens, each containing a single Alpine buck with proven libido. The buck-to-goat ratio was 1:28. The bucks were rotated between pens daily until their removal at the end of Day 42, after which all goats were merged into a single group.

After breeding and at the end of the supplementation period, all goats were managed as a single flock in a pen with sufficient space to allocate them (1.5 m^2^/head). The numbers of fetuses were determined by transabdominal ultrasonography 35–60 d after the removal of the fertile bucks. An ultrasound (Samsung-Medison SA-600 with a 4 MHz convex probe; Samsung Co., Seoul, Republic of Korea) fitted to a 4 MHz transabdominal convex probe was used. Pregnancy was confirmed by detection of products of conception (uterine fluid, placentomes, fetal membranes, and/or fetus(es)) and the fetal number was estimated based on at least 2 evaluations. Based on the data collected, the following reproductive parameters were calculated: fertility ([pregnant goats/total goats] × 100), prolificacy ([pregnant goats with twins/total pregnant goats] × 100), and reproductive rate ([total number of newborns/total goats] × 100). At kidding, the date, birth weight, and litter size of the F1 progeny were recorded. Using these data, the F0 conception date and days to conception after the introduction of the fertile bucks were estimated by subtracting 150 days (goat’s gestational length) from the F1 birth date. The goats were weighed weekly throughout the experiment to monitor daily weight gain using a mobile scale with a 200 kg capacity and a precision of 0.05 kg (Torey^®^).

### 2.4. Plasma Metabolite Profiles

Plasma samples were collected at four time points: before treatment initiation (Day–14), during treatment (Days 15 and 40), and 15 days after treatment cessation (Day 57). To minimize stress responses, all goats were well acclimated to the handling procedures associated with weekly blood sampling. Blood samples were obtained via jugular venipuncture, immediately treated with EDTA (BD Vacutainer, Preanalytical Solutions, Franklin Lakes, NJ, USA), rapidly cooled to 4 °C, and centrifuged at 3000 rpm for 15 min within 2 h of collection to obtain plasma. Plasma samples were stored at −20 °C until analysis.

Biochemical analyses were performed using commercial kits, following the manufacturer’s instructions. For all assays, quality control samples were considered acceptable if the coefficient of variation was below 10% at each level, and if replicate measurements fell within the manufacturer’s specified range. Measurements were conducted using a Mindray BA-88A semi-automatic blood chemistry analyzer, which was pre-calibrated with a calibrator and control serum levels (Spintrol normal, Reference 1002120; Spintrol Pathologic, Reference 1002211; Spintrol CAL, Reference 1002011) and commercial reagents. All kits were sourced from Spinreact (Girona, Spain). The following parameters were measured using the corresponding methods and sensitivities: total protein concentration (Reference 1001291, biuret method, 540 nm), sensitivity: 0.082 per 1 mg/dL; albumin (Reference 1001020, bromocresol green method, 630 nm), sensitivity: 0.200 per 1 mg/dL. Blood urea nitrogen (BUN) (41041, enzymatic method, 340 nm), sensitivity: 0.005 per 1 mg/dL; creatinine (1001111, alkaline picrate method, 492 nm), sensitivity: 0.047 per 1 mg/dL; bilirubin (Reference 1001044, the sulfanilic acid diazotized method, 555 nm), sensitivity: 0.026 per 1 mg/dL; cholesterol (Reference 41020, enzymatic method, 505 nm), sensitivity: 0.026 per 1 mg/dL; glucose (Reference 41011, enzymatic method, 505 nm), sensitivity: 0.004 per 1 mg/dL; calcium (Reference 1001060, o-cresolphthalein method, 570 nm), sensitivity: 0.031 per 1 mg/dL; phosphorus (Reference 1001155, ammonium molybdate method, 340 nm), sensitivity: 0.080 per 1 mg/dL; gamma-glutamyl transferase (GGT) (Reference 41292, carboxy substrate method, 405 nm), sensitivity: 0.0008 per 1 U/L; alanine aminotransferase (ALT) (Reference 41282, NADH Kinetic UV, IFCC rec. liquid method, 340 nm), sensitivity: 0.0005 per 1 U/L; aspartate aminotransferase (AST) (Reference 41272, NADH Kinetic UV, IFCC rec. liquid method, 340 nm), sensitivity: 0.0005 per 1 U/L; alkaline phosphatase (ALP) (Reference 41242, p-Nitrophenylphosphate method, 405 nm), sensitivity: 0.0003 per 1 U/L.

### 2.5. Statistical Analysis

A completely randomized design was used, and data were analyzed using the SAS statistical package, version 9.4 [39]. Individual animals were the experimental units. The data were tested for normality with PROC-UNIVARIATE (Shapiro–Wilks normality test). A prior power analysis (Proc POWER) indicated that the power of the analysis is 0.80 for an alpha level of 0.05.

BUN, creatinine, ALT, ALP, AST, albumin, GGT, total protein, bilirubin, glucose, cholesterol, phosphorus, and calcium concentrations were analyzed using linear mixed model procedures (PROC-MIXED). Treatment was the fixed effect. Live weight was included as a covariate. The sampling dates were included as repeated measures, and animal ID was included as a random effect.

Live weight at the start and end of the experiment, and average daily weight gains during the experiment, were analyzed using linear mixed model procedures (PROC-MIXED). Average daily weight gains were calculated by subtracting the final weight from the initial weight, divided by the number of experimental days. Treatment was the fixed effect.

Fertility ([pregnant goats/total goats] × 100) and prolificacy ([pregnant goats with twins/total pregnant goats] × 100) were analyzed using the generalized linear mixed model procedures with a binomial distribution and logit link function (PROC-GLIMMIX). Treatment was the fixed effect. The initial live weight was included as covariate. Data for reproductive rate ([total number of newborns/total goats] × 100) were analyzed using the generalized linear mixed model procedures with a multinomial distribution and logit link function (PROC-GLIMMIX). The same fixed effect and covariates were used for the analysis of fertility and prolificacy. The fertility, prolificacy, and reproductive rate data are presented as logit values and back-transformed percentages. Data for days to conception after buck introduction were analyzed using PROC-MIXED. The same fixed effects, covariates, and random effects were used to analyze the reproductive outcomes. Significant differences among means for the treatments for variables measured at different time points during the experiment were analyzed using the LSD of PROC GLM. The fertility and reproductive rate data are presented as logit values and back-transformed percentages. All continuous data are presented as mean ± SEM.

## 3. Results

### 3.1. Kalanchoe daigremontiana and Kidney Function

**Figure 2:**
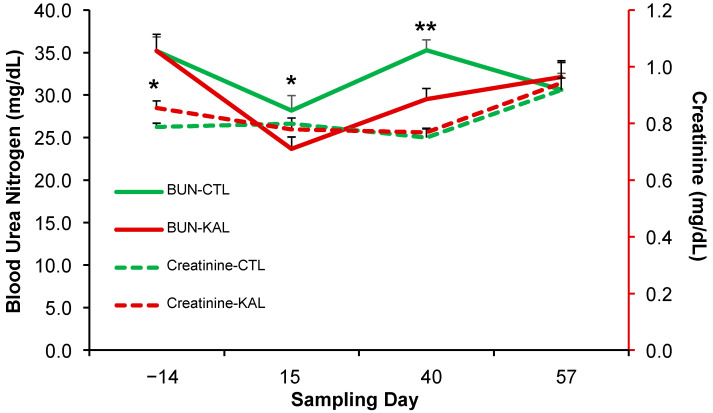
Mean (±) circulating concentrations of BUN (solid line) and creatinine (dashed line) in multiparous Alpine goats supplemented with *Kalanchoe daigremontiana* (red lines) or without supplementation (green lines) for 42 d during the breeding period. * *p* < 0.05; ** *p* < 0.01.

Circulating BUN concentrations differed significantly between treatments on Day 15 (*p* < 0.05) and Day 40 (*p* < 0.01) of the sampling period, with higher values observed in the CTL group (Figure 2). No significant differences were detected on the other sampling dates (*p* > 0.05). Overall, circulating BUN concentrations remained comparable between treatments over time (*p* > 0.05). Circulating creatinine concentrations differed significantly between treatments on Day 14 of the sampling period, with higher values in the KAL group (*p* < 0.05; Figure 2). However, no significant differences were observed on subsequent sampling dates (*p* > 0.05). Across the entire study period, creatinine concentrations remained similar between treatments (*p* > 0.05).

### 3.2. Kalanchoe daigremontiana and Livcr Function

**Figure 3:**
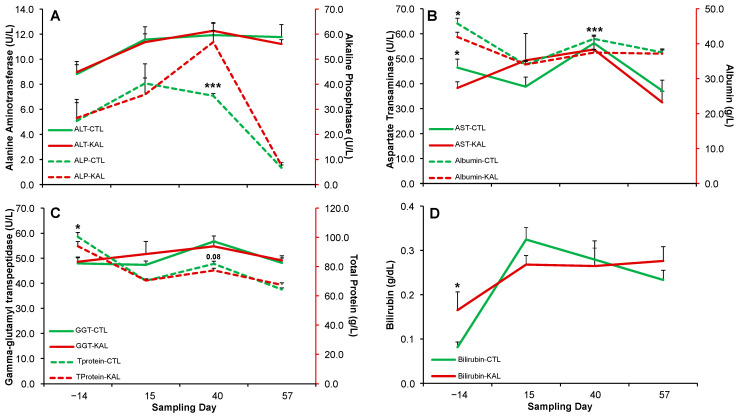
Mean (±) circulating concentrations of ALT ((**A**)—solid line), ALP ((**A**)—dashed line), AST ((**B**)—solid line), albumin ((**B**)—dashed line), GGT ((**C**)—solid line), total protein ((**C**)—dashed line), and bilirubin ((**D**)—solid line) in multiparous Alpine goats supplemented with *Kalanchoe daigremontiana* (red lines) or without supplementation (green lines) for 42 d during the breeding period. * *p* < 0.05; *** *p* < 0.001.

Circulating ALT concentrations progressively increased over time; however, no significant differences were observed between treatments at any sampling date (*p* > 0.05; Figure 3A) or across time (*p* > 0.05). Circulating ALP concentrations increased initially but declined sharply after the breeding period (Figure 3A). A significant difference was detected between treatments on Day 40 of the sampling period, with higher values in the KAL group (*p* < 0.001; Figure 3A), but not on other sampling dates (*p* > 0.05) or across time (*p* > 0.05). Circulating AST concentrations fluctuated over time (Figure 3B) but showed no significant differences across time (*p* > 0.05). However, AST concentrations were significantly higher in the CTL group on Day 14 of the sampling period (*p* < 0.05; Figure 3B), with no differences observed on subsequent dates (*p* > 0.05). Circulating albumin concentrations decreased over time (Figure 3B) but did not significantly differ across time (*p* > 0.05). However, albumin levels were significantly higher in the CTL group on Day 14 (*p* ≤ 0.05) and Day 40 sampling dates (*p* < 0.001; Figure 3B), with no differences detected on other dates (*p* > 0.05). Circulating GGT concentrations increased slightly over time (Figure 3C) but did not differ significantly between treatments at any sampling date or across time (*p* > 0.05). Circulating total protein concentrations declined over time (Figure 3C) but remained statistically similar across time (*p* > 0.05). However, total protein levels were significantly higher in the CTL group on the Day 40 of the sampling period (*p* ≤ 0.05) and showed a trend toward significance on the Day 57 of the sampling period (*p* = 0.08; Figure 3C), with no differences observed on other dates (*p* > 0.05). Circulating bilirubin concentrations increased over time (Figure 3D) but did not significantly differ across time (*p* > 0.05). The bilirubin levels were significantly higher in the KAL group on Day 14 of the sampling period (*p* < 0.05) but showed no differences on subsequent dates (*p* > 0.05).

### 3.3. Kalanchoe daigremontiana and Metabolites and Minerals

**Figure 4:**
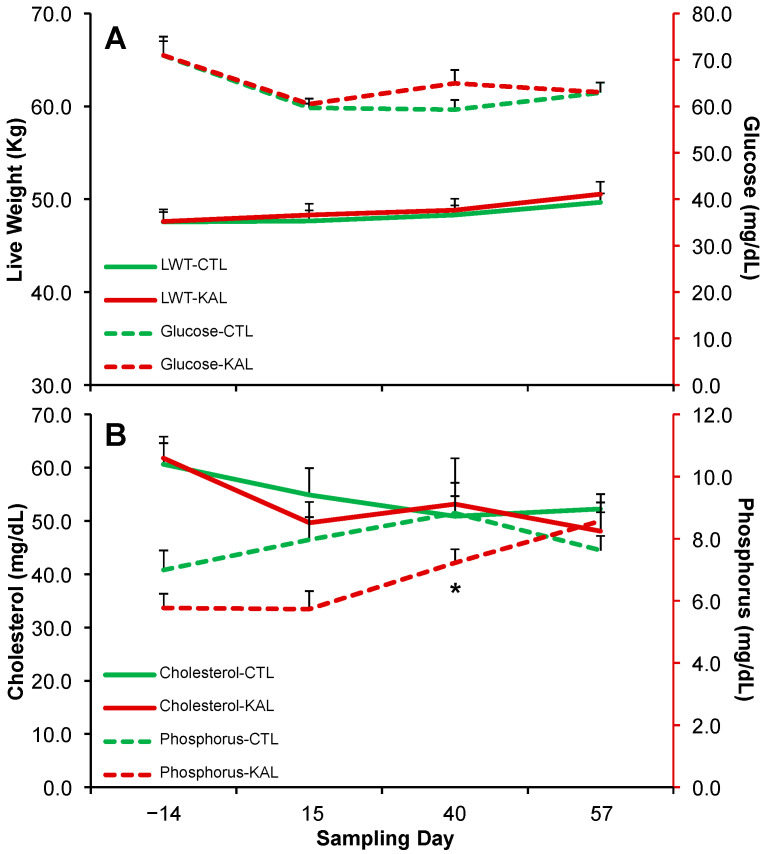
Mean (±) live weight ((**A**)—solid line), glucose ((**A**)—dashed line), cholesterol ((**B**)—solid line), and phosphorus ((**B**)—dashed line) in multiparous Alpine goats supplemented with *Kalanchoe daigremontiana* (red lines) or without supplementation (green lines) for 42 d during the breeding period. * *p* < 0.05.

Circulating glucose (Figure 4A) and cholesterol (Figure 4B) concentrations in goats slightly decreased over time. However, no significant differences were observed between treatments at any sampling date (*p* > 0.05) or across time (*p* > 0.05). Circulating phosphorus concentrations increased until the end of the breeding period, after which they decreased. A significant difference was observed between treatments on Day 40 of the sampling period, with higher values in the CTL group (*p* < 0.01; Figure 4B), but no differences were found on other sampling dates (*p* > 0.05). Overall, phosphorus concentrations were similar between treatments across time (*p* > 0.05; Figure 4B). Circulating calcium concentrations slightly increased over time (from an average of 6.6 to 8.6 mg/dL). However, no significant differences were observed between treatments at any sampling date (*p* > 0.05) or across time (*p* > 0.05).

### 3.4. Kalanchoe daigremontiana and Body Weight Variables

At enrollment, the average body weight of goats in the KAL and CTL treatments was 47.6 ± 1.3 kg and 47.6 ± 1.1 kg, respectively (*p* > 0.05; Figure 4A). The average daily live weight gain was 0.61 ± 0.14 kg for the KAL group and 0.36 ± 0.08 kg for the CTL group, with no significant difference between treatments (*p* > 0.05). The final body weight was 48.8 ± 1.2 kg for the KAL group and 48.3 ± 1.1 kg for the CTL group, with no significant difference between treatments (*p* > 0.05; Figure 4A).

### 3.5. Kalanchoe daigremontiana and Reproductive Performance

In total, 95% of females were pregnant, with 75% carrying twins, leading to a reproductive rate of 165%. Of the pregnant females, a high percentage (67%) were pregnant in their first reproductive cycle after introducing the fertile buck, and the rest (33%) were pregnant in their second reproductive cycle.

**Figure 5:**
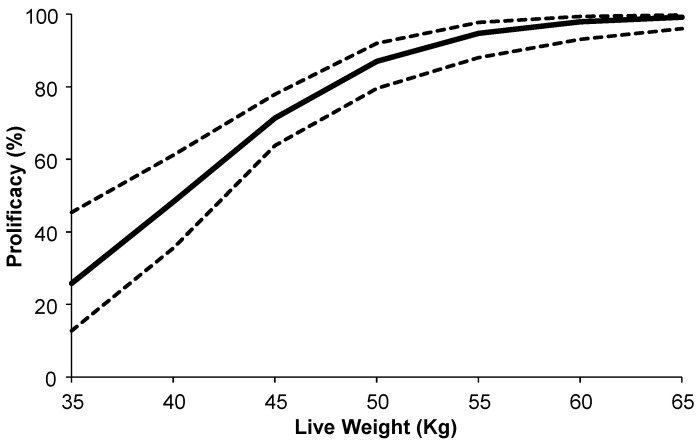
Relationship between live weight at the start of breeding and prolificacy of multiparous Alpine goats, with the KAL and the CTL goats combined. The dashed lines represent upper and lower 95% confidence limits.

The conception date differed between treatments. The females from the KAL treatment were pregnant on average 13 d after the introduction of the buck, whereas the females from the CTL treatment were pregnant on average 19 d after the introduction of the buck (*p* < 0.05). The conception date was not influenced (*p* > 0.05) by live weight at the beginning of the breeding period. Fertility (KAL: 93% vs. CTL: 96%), prolificacy (KAL: 77% vs. CTL: 73%), and reproductive rate (KAL: 164% vs. CTL: 167%) were similar between treatments (*p* > 0.05). Prolificacy (*p* < 0.01; Figure 5) and reproductive rate (*p* < 0.05) were influenced by live weight at the beginning of the breeding period, but not fertility rate (*p* > 0.05). The relationship between prolificacy and live weight at the start of the breeding period was curvilinear (Figure 5). The regression analysis estimated an average of 12% increase in prolificacy rate for every 5.0 kg increase in live weight at the start of the breeding period (Figure 5).

## 4. Discussion

This study examined the effects of *K. daigremontiana*, a nutrient-rich plant, on key physiological parameters in goats, focusing on kidney and liver function markers, metabolic indicators, and reproductive performance. The analysis of these biomarkers provides valuable insights into the potential benefits and risks of supplementing *K. daigremontiana* in goat diets, particularly given its documented toxicological effects in other animal species. While many biochemical parameters did not show significant differences between treatments, specific instances were observed where *K. daigremontiana* supplementation had a measurable impact on goat physiology. To the best of our knowledge, this plant has not previously been incorporated into goat diets, and optimal experimental dosages have yet to be established. Further research is necessary to determine appropriate dosing strategies and evaluate its effects at different stages of the goat production cycle.

### 4.1. Kalanchoe daigremontiana and Kidney Function

We hypothesized that supplementing a goat’s diet with *K. daigremontiana* would enhance kidney function. Blood urea nitrogen (BUN) and creatinine concentrations are key biomarkers of renal function and indicate protein metabolism [40,41]. In our study, the BUN concentrations fluctuated but remained within the reference range for healthy goats [42]. However, goats receiving the *K. daigremontiana* (KAL) treatment exhibited significantly lower BUN concentrations on Days 15 and 40 of the sampling period, suggesting a potential influence on protein metabolism or renal filtration processes. Since BUN is primarily excreted by the kidneys, its reduction in KAL-treated goats may indicate improved renal function or altered nitrogen metabolism. Conversely, the increase in BUN concentration observed in the control (CTL) group may reflect enhanced protein catabolism, potentially associated with kidney dysfunction or a lower-quality diet [43]. Nonetheless, the similarity in BUN concentrations across other time points suggests that the effects of *K. daigremontiana* on kidney function may be transient or influenced by additional factors such as diet composition or physiological stress [43,44].

Creatinine levels are a key indicator of renal filtration efficiency, and the concentrations observed in this experiment remained within the reference range for healthy goats [42]. We noted an overall increase in creatinine concentrations toward the end of the experiment in both treatment groups. However, on Day 14 of the sampling period, creatinine levels were significantly higher in *K. daigremontiana* (KAL)-treated goats, after which they stabilized and became similar across treatments. This initial elevation may reflect an early alteration in kidney function, as the sample was collected before *K. daigremontiana* was introduced into the diet. In contrast to our findings, Tharwat et al. [45] reported decreased creatinine concentrations in goats transitioning from gestation to lactation. This discrepancy between pregnant goats [45] and the non-pregnant goats in our study may be attributed to differences in physiological state, such as the increasing fasting body weight of goats in our experiment [46]. Similar to BUN, the transient fluctuations in creatinine concentrations could indicate an initial metabolic response or adaptation to *K. daigremontiana*, which contains bioactive compounds such as bufadienolides, which may influence renal function [10,21,47,48]. The absence of sustained differences over time suggests that any potential renal stress or alterations in kidney function were short-lived, supporting the notion that moderate exposure may not result in chronic renal impairment or toxicity.

From a practical perspective, these findings suggest that moderate inclusion of *K. daigremontiana* in goat diets is unlikely to compromise renal function, as neither BUN nor creatinine levels showed sustained deviations indicative of kidney impairment. However, the transient fluctuations observed in these biomarkers underscore the need for further research to better understand the short-term metabolic responses to dietary changes and the potential influence of bioactive compounds in *K. daigremontiana*.

### 4.2. Kalanchoe daigremontiana and Liver Function

Regarding liver function, we hypothesized that supplementing a goat’s diet with *K. daigremontiana* would enhance hepatic function. Liver enzymes, including ALT, ALP, AST, and GGT, are key indicators of liver health and metabolic activity [49,50]. In this experiment, the observed enzyme activity levels remained within the reference range for healthy goats [42]. Our results showed no significant differences in ALT and GGT concentrations between treatments throughout the sampling period. Similarly, Djuricic et al. [51] reported no differences in ALT and GGT activity between primiparous and multiparous pregnant goats of two genotypes (Boer and Saanen) during the puerperal period. ALT levels typically increase in response to liver damage; in the absence of hepatic injury, the ALT and GGT activity remains stable [52]. Consequently, our findings suggest that *K. daigremontiana* supplementation did not significantly affect ALT and GGT enzyme activity, indicating no apparent impact on liver function.

ALP concentrations were higher in *K. daigremontiana* (KAL)-treated goats on Day 40, suggesting a transient hepatic response or a metabolic shift induced by dietary supplementation. Similarly, Yáñez-Ruiz and Molina-Alcaide [53] reported increased ALP activity in non-pregnant Granadina goats following the incorporation of olive leaves into their diet, indicating that specific dietary compounds may influence liver function. However, conflicting reports exist regarding the effects of both intrinsic and extrinsic factors on ALP activity in goats [54,55]. The liver plays a central role in energy metabolism in goats [56,57]. In our study, a higher proportion of females conceived during their first reproductive cycle following male introduction. The elevated ALP concentrations observed in KAL-treated goats may be associated with increased energy demand for implantation [58]. Although elevated ALP is often considered a marker of liver dysfunction [49,50], the absence of significant changes over time and the transient nature of this effect suggest that these fluctuations are more likely physiological rather than pathological. AST concentrations varied throughout this study but were significantly lower in KAL goats on Day 14 of the sampling period, after which they stabilized and became comparable across treatments. Since AST is present in both liver and muscle tissues [59,60], this initial difference may reflect an early alteration in hepatic or muscular function. However, as the first sample was collected before *K. daigremontiana* was introduced into the diet, this variability is unlikely to be a direct response to the treatment. The lack of significant differences in subsequent samples suggests that any early metabolic shifts had normalized over time. Overall, while some differences in ALP, AST, and GGT activity were observed, they were inconsistent across sampling dates, indicating that *K. daigremontiana* supplementation did not exert a substantial long-term impact on liver function.

Albumin, a key marker of liver protein synthesis, was higher in *K. daigremontiana* (KAL)-treated goats on Days 14 and 40 of the sampling period. As a vital protein, albumin plays a crucial role in regulating blood volume, maintaining osmotic pressure, and transporting various biomolecules [51]. Elevated albumin concentrations may indicate a favorable metabolic response to *K. daigremontiana*, potentially reflecting enhanced protein metabolism or liver function. However, the absence of significant differences over time suggests that this effect was transient. The lack of sustained alterations further supports the notion that moderate *K. daigremontiana* supplementation does not lead to chronic hepatic impairment or toxicity.

### 4.3. Kalanchoe daigremontiana and Metabolites and Minerals

Regarding metabolites and minerals, we hypothesized that supplementing a goat’s diet with *K. daigremontiana* would enhance metabolites and minerals, including glucose, cholesterol, calcium, and phosphorus levels. These biomarkers were assessed to evaluate the overall physiological effects of *K. daigremontiana* supplementation. Although slight decreases in circulating glucose and cholesterol concentrations were observed over time, no statistically significant differences were detected between treatments, suggesting that supplementation did not substantially impact metabolite markers. Notably, glucose and cholesterol concentrations remained within the reference range for healthy adult goats [61,62]. In rodent models, oral administration of a *K. pinnata* extract at doses of 10 mg/kg [63], 60 mg/kg [64], and 500 mg/kg [65] resulted in reductions in glucose, cholesterol, and triglyceride levels, suggesting a potential hypoglycemic effect. The results observed in the mouse model may be attributed to the faster metabolism of monogastric animals compared to ruminants. However, whether *K. daigremontiana* exerts similar metabolic effects in goats remains to be determined.

Similarly, although calcium levels increased slightly over time, no significant differences were observed between treatments, indicating that *K. daigremontiana* supplementation did not disrupt mineral homeostasis. However, phosphorus levels were significantly higher in CTL goats on Day 40 of the sampling period, suggesting potential differences in mineral absorption or metabolism between treatments. Despite this transient variation, phosphorus concentrations remained comparable between groups throughout this study period, indicating that *K. daigremontiana* did not cause long-term disruptions in mineral balance.

### 4.4. Kalanchoe daigremontiana Body Weight and Reproductive Performance

No significant differences in body weight were observed between the KAL and CTL treatments at enrollment, during the breeding period, or at the conclusion of the experiment. This suggests that *K. daigremontiana* supplementation did not positively or negatively affect goat growth, as both groups exhibited similar live weight changes. In contrast to our findings, supplementation with *K. daigremontiana* in Cobb chickens improved feed conversion efficiency and resulted in greater final body weight [30]. Once again, variations in the metabolic absorption rate in ruminants and the dosage and duration of supplementation may have influenced the results.

Reproductive performance was similar between treatments, with both groups exhibiting high pregnancy rates (95%) and prolificacy (~75% twin births). This reproductive efficiency is typical in well-nourished and multiparous females [15,66]. Nevertheless, the KAL treatment was associated with an earlier conception date, as KAL females conceived, on average, 13 days after buck introduction, compared to 19 days in the CTL group. This suggests that *K. daigremontiana* supplementation may enhance reproductive efficiency or stimulate earlier estrus cycles, though it did not significantly affect fertility, prolificacy, or overall reproductive success. Despite these findings, the use of *K. daigremontiana* in livestock diets remains limited, and further research is needed to determine the optimal supplementation level that maximizes productive and reproductive benefits while minimizing potential toxicity risks.

### 4.5. Strengths and Limitations

The strength of the present study lies in its well-structured design, clearly defined research question, and comprehensive evaluation of key biomarkers, providing a holistic assessment of the potential effects of *K. daigremontiana* on animal health and productivity. However, we acknowledge existing knowledge gaps and emphasize the need for further research to optimize the plant’s dosage, ensuring both efficacy and safety while minimizing potential toxicity risks. Several limitations emerged from this experiment, emphasizing the urgency of addressing these knowledge gaps. One limitation is the variability observed in several measured parameters over time, with no sustained differences between treatments. While this may reflect an adaptive or transient physiological response, it also limits the generalizability and robustness of our conclusions. Despite controlled and similar conditions for both treatment groups, potential confounding factors, such as diet composition and environmental stressors, may have influenced kidney and liver biomarker fluctuations, potentially obscuring the plant’s actual effects. Additionally, the dosage used in this study may have been insufficient to elicit a measurable response in goats, whose slower metabolic rate, compared to that of monogastric animals, could affect toxin absorption and metabolism. *K. daigremontiana* contains known cardiotoxins, yet no detectable toxin residues or metabolites were observed in this experiment. This raises concerns about potential limitations in detection methods or experimental conditions and highlights the need for further investigation into these compounds’ degradation, transformation, or bioavailability. These factors are critical for assessing safety risks and ensuring accurate toxicological evaluations. Moreover, it is possible that the additive was not effectively absorbed or did not interact with the digestive system in a manner that produced a detectable physiological effect. Given the unique dietary adaptations and metabolic processes of goats, extrapolating these findings to other livestock species remains challenging and warrants further investigation.

## 5. Conclusions

This study offers valuable insights into the effects of *K. daigremontiana* on kidney and liver function, overall health status, and reproductive performance in goats. While the observed effects were often transient or limited to specific time points, this study suggests potential benefits, particularly in enhancing renal function and influencing metabolic processes associated with liver enzymes. However, the lack of consistent differences over time and the overall similarity in reproductive and metabolite markers between treatments indicate that *K. daigremontiana* supplementation exerts a transient effect rather than a sustained impact on goat health. Although the findings are promising, further research is needed to investigate the long-term effects and optimal dosages of *K. daigremontiana* on metabolic processes and animal performance.

## Figures and Tables

**Table 1:** Ingredients and nutrient composition (DM basis) of the balanced isoproteic and isoenergetic diet provided during the experiment.

	*K. daigremontiana* (kg/ton)	
Ingredient	0	2
Alfalfa (%)	65	64.9
Oat hay (%)	15	15
Maize silage (%)	20	19.9
*K. daigremontiana* (%)	0	0.2
Chemical composition		
Dry matter (%)	80.3	80.4
Crude protein (%)	13.4	13.4
ME (Mcal/kg/DM)	2.42	2.41

## Data Availability

The data presented in the manuscript are not deposited in an official repository but can be made available upon reasonable request.
